# Worse pulmonary function in association with cumulative exposure to nanomaterials. Hints of a mediation effect via pulmonary inflammation

**DOI:** 10.1186/s12989-024-00589-3

**Published:** 2024-06-28

**Authors:** Giulia Squillacioti, Thomas Charreau, Pascal Wild, Valeria Bellisario, Federica Ghelli, Roberto Bono, Enrico Bergamaschi, Giacomo Garzaro, Irina Guseva Canu

**Affiliations:** 1 Department of Public Health and Pediatrics, University of Turin, Via Santena 5 bis, 10126 Torino, Italy; 2 Department of Occupational and Environmental Health, Center for Primary Care and Public Health (Unisanté), University of Lausanne, Epalinges, Lausanne, 1066 Switzerland; 3 Città della Salute e della Scienza di Torino, University Hospital, Via Zuretti 29, 10126 Turin, Italy

**Keywords:** Nanomaterials, Exhaled breath condensate, Pulmonary function, Spirometry, Inflammation, Biomarkers, Public health, Occupational health

## Abstract

**Background:**

Today, nanomaterials are broadly used in a wide range of industrial applications. Such large utilization and the limited knowledge on to the possible health effects have raised concerns about potential consequences on human health and safety, beyond the environmental burden. Given that inhalation is the main exposure route, workers exposed to nanomaterials might be at risk of occurrence of respiratory morbidity and/or reduced pulmonary function. However, epidemiological evidence regarding the association between cumulative exposure to nanomaterials and respiratory health is still scarce. This study focused on the association between cumulative exposure to nanomaterials and pulmonary function among 136 workers enrolled in the framework of the European multicentric NanoExplore project.

**Results:**

Our findings suggest that, independently of lifelong tobacco smoking, ethnicity, age, sex, body mass index and physical activity habits, 10-year cumulative exposure to nanomaterials is associated to worse FEV_1_ and FEF_25 − 75%_, which might be consistent with the involvement of both large and small airway components and early signs of airflow obstruction. We further explored the hypothesis of a mediating effect via airway inflammation, assessed by interleukin (IL-)10, IL-1β and Tumor Necrosis Factor alpha (TNF-α), all quantified in the Exhaled Breath Condensate of workers. The mediation analysis results suggest that IL-10, TNF-α and their ratio (i.e., anti-pro inflammatory ratio) may fully mediate the negative association between cumulative exposure to nanomaterials and the FEV_1_/FVC ratio. This pattern was not observed for other pulmonary function parameters.

**Conclusions:**

Safeguarding the respiratory health of workers exposed to nanomaterials should be of primary importance. The observed association between cumulative exposure to nanomaterials and worse pulmonary function parameters underscores the importance of implementing adequate protective measures in the nanocomposite sector. The mitigation of harmful exposures may ensure that workers can continue to contribute productively to their workplaces while preserving their respiratory health over time.

**Supplementary Information:**

The online version contains supplementary material available at 10.1186/s12989-024-00589-3.

## Introduction

According to the revised definition of the European Commission, a nanomaterial is “*a natural, incidental or manufactured material consisting of solid particles that are present, either on their own or as identifiable constituent particles in aggregates or agglomerates, and where 50% or more of these particles in the number-based size distribution fulfill at least one of the following three conditions: (a) one or more external dimensions of the particle are in the size range 1 nm to 100 nm; (b) the particle has an elongated shape, such as a rod, fiber or tube, where two external dimensions are smaller than 1 nm and the other dimension is larger than 100 nm; (c) the particle has a plate-like shape, where one external dimension is smaller than 1 nm and the other dimensions are larger than 100 nm*” [1]. Due to their small size, nanomaterials exhibit unique properties compared to their bulk counterparts including high surface area-to-volume ratio, and quantum effects. They can be classified into different types based on dimensions, such as nanoparticles, nanorods/nanowires, nanosheets, and nanotubes [2]. Today, nanomaterials are utilized in a wide range of industrial applications due to their unique properties and functionalities. Some of the principal industrial uses of nanomaterials include electronics, medicine, healthcare and cosmetic, energy storage and conversion, water and air purification, agriculture, food processing and preservation, coatings and surfaces, textiles, aerospace and automotive [3]. Such a large utilization of nanomaterials and the limited knowledge relating to the possible health effects due to exposure to ever-emerging products have also raised some concerns regarding their impact on human health and safety, beyond the environmental burden. The lack of systematic studies on hazards related to the exposure to nanomaterials has led several scientific consortia to extensive research on the potential mechanisms of toxicity and early biological adverse effects such as oxidative stress, genotoxicity and inflammation [4].

Current evidence on nanomaterials toxicity is mostly derived from experimental studies, which are challenging to translate into human health risks. Within the project “NanoExplore”, supported by the European Commission LIFE program [Grant LIFE17 ENV/GR/000285], aimed at addressing the health effects of occupational exposure to nanomaterials by launching an international prospective cohort study [5] we collected and analyzed the first data. As previously reported in [6], we found a positive dose-response relationship between exposure to nanomaterials, measured as particle number concentration and Lung-Deposited Surface Area (LDSA), and concentrations of inflammatory biomarkers, namely interleukin (IL)-10, IL-1β and Tumor Necrosis Factor (TNF)-α, measured in exhaled breath condensate (EBC). We also found a negative relationship with both dose-metrics with the Total Antioxidant Potential measured in urine of workers handling nanomaterials. This first study suggested that current occupational exposure to nanomaterials can be associated with local inflammatory mechanisms. Consistently, previous authors have reported that inflammatory biomarkers, including TNF-α and ILs, were higher in biofluids from workers exposed to pigment-grade TiO_2_ [7], nanocomposites [8], nano-TiO_2_ [9], multi-walled carbon nanotubes [10] or nanoscale carbon black [11], while some authors reported no association [12]. To date, the evidence upon the association between exposure to nanomaterials and respiratory health is very limited. Given that inhalation is the main exposure route for nanomaterials [13, 14], exposed workers might be at higher risk of occurrence of respiratory morbidities [4, 11], changes in cardiopulmonary function [9, 15] and/or reduced pulmonary function [8, 11, 16]. Furthermore, the harmful effects of the exposure to nanomaterials might be mediated by airway inflammation [17]. The study of inflammatory mediators in EBC may provide a valuable piece of information on early biological effects occurring at the pulmonary level in response to external stimuli, including air pollution and work-related airborne exposures [18, 19]. However, the lack of standardization and clinical validation with established reference intervals for biomarkers of early effects that could be measured in EBC have limited their usage in the conventional workplace health surveillance [20]. Although, the non-invasive nature of EBC sampling and its supposed ability to mirror lung bio-pathology [21] make it a promising tool for human biomonitoring, clinical and surveillance purposes, as well as epidemiological studies [22].

In light of the aforementioned considerations, the aim of the present study is to evaluate, in the NanoExplore workers, whether there is an association between cumulative exposure to nanomaterials and respiratory function parameters, accounting for potential confounders and mediators. In this concern, as an additional aim, we also explored the potential mediating role of inflammatory biomarkers measured in EBC. The identification of such associations will serve as the starting point for developing models enabling describing the cause-effect relationship between the exposure to nanomaterials, the inflammatory biomarkers and the respiratory function.

## Materials and methods

### Study design and participants

Current analyses are based on data acquired in the framework of the open multicenter prospective cohort study “NanoExplore”. NanoExplore aims to improve the understanding of levels, nature and possible adverse health effects associated with exposure to nanomaterials in indoor workplaces and urban areas. The project uses a holistic approach to integrate human biomonitoring with measured environmental data on exposure to nanomaterials for supporting the development of future risk management guidelines. More details on the harmonized protocol [5], sample and exposure description as well as the first results are presented elsewhere [6, 23, 24].

Briefly, the study population included adult workers of both sexes pre-identified as potentially exposed or unexposed to nanomaterials during the preparatory company visits. The study was multicentric and involved seven centers located in Italy, Spain and Switzerland. The participating companies were enrolled based on a confirmed prior knowledge of their activities related to nanomaterials. The minimal sample size was estimated a priori and only pre-identified exposed and unexposed workers were invited to participate to meet the sample requirements [5]. All workers who provided a written informed consent to participate were included. The final study sample consists of workers who provided biological samples at the beginning and at the end of the field campaign, for whom individual exposure estimates and lung function measurements were available (Figure S1). Ethics approval has been obtained from the local Ethics Committees: The Swissethics in Switzerland (approval 2020 − 01098); the Bio-ethical Committee of the University of Torino in Italy (approval 336,577 8.08.2020); and the Health and Safety Board of the Catalan Institute of Nanoscience and Nanotechnology, in Spain (approval ICN2-22-03-2022). All workers provided a written consent to participate in this study.

### Cumulative exposure to nanomaterials

Current exposure to nanomaterials was measured using the portable nanoparticle counters “DiSCmini™” (Testo, Mönchaltorf, Switzerland) placed in close proximity to the workstations for a minimum of two to a maximum of four consecutive working days. The DiSCmini measures the particle number concentration and the average diameter of nanoparticles with a time resolution down to 1 s (1 Hz) and provides the particle number concentration, expressed as number of particles/cm^3^, and the LDSA (µm^2^/cm^3^), corresponding to the probability of particle deposition in the tracheobronchial and alveolar regions of the lung [25]. The detection range is 500-1,000,000 particles/cm^3^ for particles with an aerodynamic diameter ranging from 10 to 300 nm. Petremand et al. showed the relevance of using DiSCmini for ultrafine particle monitoring [26] and provide some practical statements on how a combination of particle detection devices based on different physical principles – OPC such as Environmental Dust Monitoring (GRIMM, EDM1.109) – should be analyzed to provide a reliable estimation of the aerosol number concentration over the largest range of interest [27].

Since most participants had a complex occupational history, we decided to standardize the estimation of the cumulative exposure by limiting it to the last 10 years. A similar strategy is regularly applied in occupational epidemiology [28, 29]. We derived the cumulative occupational exposure over the last 10 years by multiplying the particle number or LDSA concentrations, measured by the DiSCmini, by the self-reported job duration (in years), and then rescaling it to interquartile range (IQR) as follows. First, the concentrations measured during the field campaigns were averaged and assumed to be representative of the last working year. Secondly, we multiplied the annual concentration by the years of occupation to obtain the cumulative exposure dose (particles/cm^3^-years and µm^2^/cm^3^-years, for particle number concentration and LDSA, respectively). For workers with a career shorter than 10 years, we adopted a more conservative approach by considering the years working in previous/unknown locations equal to the averaged background exposure. The background exposure was the averaged exposure assessed at the companies not handling/producing nanomaterials. If the participant worked for over 10 years at the same workstation, the duration was limited to 10 years. Finally, the cumulative exposure dose was rescaled using the IQR of the distribution. All the aforementioned calculations were adopted to derive an additional cumulative exposure over 20 years, then used for sensitivity analyses.

### Pulmonary function parameters

Spirometry test was performed, at the beginning (pre-shift) of the exposure monitoring campaign, according to the ATS/ERS standards [30] as follows. To minimize the inter-operator variability, a medical doctor, adequately trained for spirometry, assessed pulmonary function parameters using a USB-pneumotachograph (microQuark, COSMED, Agrate Brianza, Italy) in all the recruiting centers. Mean Forced Expiratory Volume in 1 s (FEV_1_), Forced Vital Capacity (FVC) and the Forced Expiratory Fluxes at 25% and 75% of FVC (FEF_25–75%_) were obtained from the three best acceptable test values of each participant, according to the ATS/ERS standards [30].

We calculated the predicted values of FEV_1_, FVC, FEV_1_/FVC and FEF_25 − 75%_ by applying standardized equations from the Global Lung Initiative (GLI) [31] enabling the comparison of the measured spirometry values with spirometric reference equations (SRE) derived from healthy individuals of the same ethnicity, height, age, and sex. For this purpose, we used the R-library “rspiro” and in case of doubt or ambiguity on the participant’s ethnicity we classified it as “other”. The GLI equations provide the lower limit of normal (LLN) values, defined as the fifth percentile (or a z-score < − 1.64) of the GLI SRE distribution of each pulmonary function parameter for the healthy non-smoking population. We compared the observed values with the LLN to determine if the parameter would belong to the lower 5% of the GLI SRE distribution and created a dichotomous variable (i.e., < LLN versus ≥ LLN).

### EBC sampling and biomarkers quantification

Two EBC samples, at the beginning (pre-shift) and at the end of the exposure monitoring campaign (post-shift), were collected using a portable collection device (Turbo-DECCS™, Medivac, Parma, Italy) and according to recommendations provided by ATS/ERS Task Force [32]. Each worker breathed tidally in a disposable mouthpiece for around 15 min to provide 2–3 mL of sample, subsequently normalized by the volume of exhaled air assessed by a flow meter (VOLMET™ 20 Medivac, Parma, Italy). EBC samples were immediately aliquoted and stored at -20 °C to minimize their degradation during the transportation to the laboratory in charge of the processing of the biological samples, where they were stored at -80 °C until analysis.

A panel of several biomarkers was measured in EBC to assess oxidative/nitrosative stress, early fibrosis and inflammation at pulmonary level, as described elsewhere [5]. Based on our previous results [6] highlighting a consistent positive relationship between exposure to nanomaterials and IL-1β, IL-10 and TNF-α, we limited current analyses on these three inflammatory biomarkers. Real time Polymerase chain reaction-enzyme linked immunosorbent assays (PCR-ELISAs) were used to quantify IL-1β, IL-10 and TNF-α (A35574, A35590 and A35601 – Invitrogen, Thermo Fisher Scientific MA, USA).

### Epidemiological questionnaire and covariates

Data from a self-administered, web-based epidemiological questionnaire were collected and managed using the Research Electronic Data Capture (REDCap) software hosted at Unisanté [33, 34]. We collected variables potentially included in the causal chain between exposure and outcome such as individual characteristics (e.g., sex, age, ethnicity, morphological parameters), tobacco smoking, household exposure to particles and lifestyle-related features (e.g., active commuting, physical activity, etc.).

The Body Mass Index (BMI) was calculated using self-reported height and weight and according to the following equation: BMI = Weight (kg) / Squared height (m^2^).

The years of smoking as well as the daily tobacco consumption declared by each participant were used to estimate the lifetime tobacco exposure expressed as pack-years, by multiplying the average daily consumption (derived by the cigarettes/pipes/cigars per day) by the duration of smoking (in years).

We created a synthetic lifestyle variable to classify workers as active or inadequately active accounting for the combination of the following variables: commuting mode, commuting time, physical activity/sport during the spare time and hours per day engaging in sedentary activities. Participants were considered active if they declared walking or cycling as commuting mode for at least 15 min each route or being engaged in physical activity/sport for at least twice a week and spending less than 9 h per day being sedentary [35]. This recoding was based on the World Health Organization guidelines on physical activity for adults [36] and on existing evidence from previous studies on the effect of physical activity on lung function [37].

### Statistical analysis

Categorical variables are reported as absolute and relative frequencies, while continuous ones are presented as mean and Standard Deviation (SD), unless differently indicated.

The percentage of missing data ranged from zero to 2%, for some general variables (age, sex, BMI, ethnicity, smoking habits – i.e., pack-years, and physical activity – i.e., lifestyle) to as high as 16%, for cumulative exposure variables to nanomaterials (expressed as particle number concentration and LDSA). To deal with missing data, we applied two procedures, one at a time: (1) a multiple imputation (MI) (STATA “mi” command) with all variables included in the final multivariable model plus one auxiliary variable (i.e., job duration), which was highly correlated with the other MI variables, generating 10 imputed datasets with 100 Markov Chain Monte Carlo (MCMC) burn-in iterations; (2) a single imputation on the exposure variable only, by replacing missing data with the center- and group-specific average of environmental exposure to nanomaterials. Indeed, we replaced the missing exposure value with the averaged exposure level of the workers from the same center and performing the same work task as workers with the missing value. All workers with missing exposure data (*n* = 22, 16% of the sample) were not involved in nanomaterial-handling procedures, yet were employed in companies from the nanocomposite sector. Therefore, they might be accidentally exposed to nanomaterials, although to low or negligible levels. Since the MI produced unrealistic higher estimates of exposure for this group of workers (a 3-fold higher median value of exposure), we opted for a more conservative approach and based our main analysis on data derived from single imputation, rather than MI. Results from MI-based models (Table S1) are in line with the main results herein presented.

**Fig. 1:**
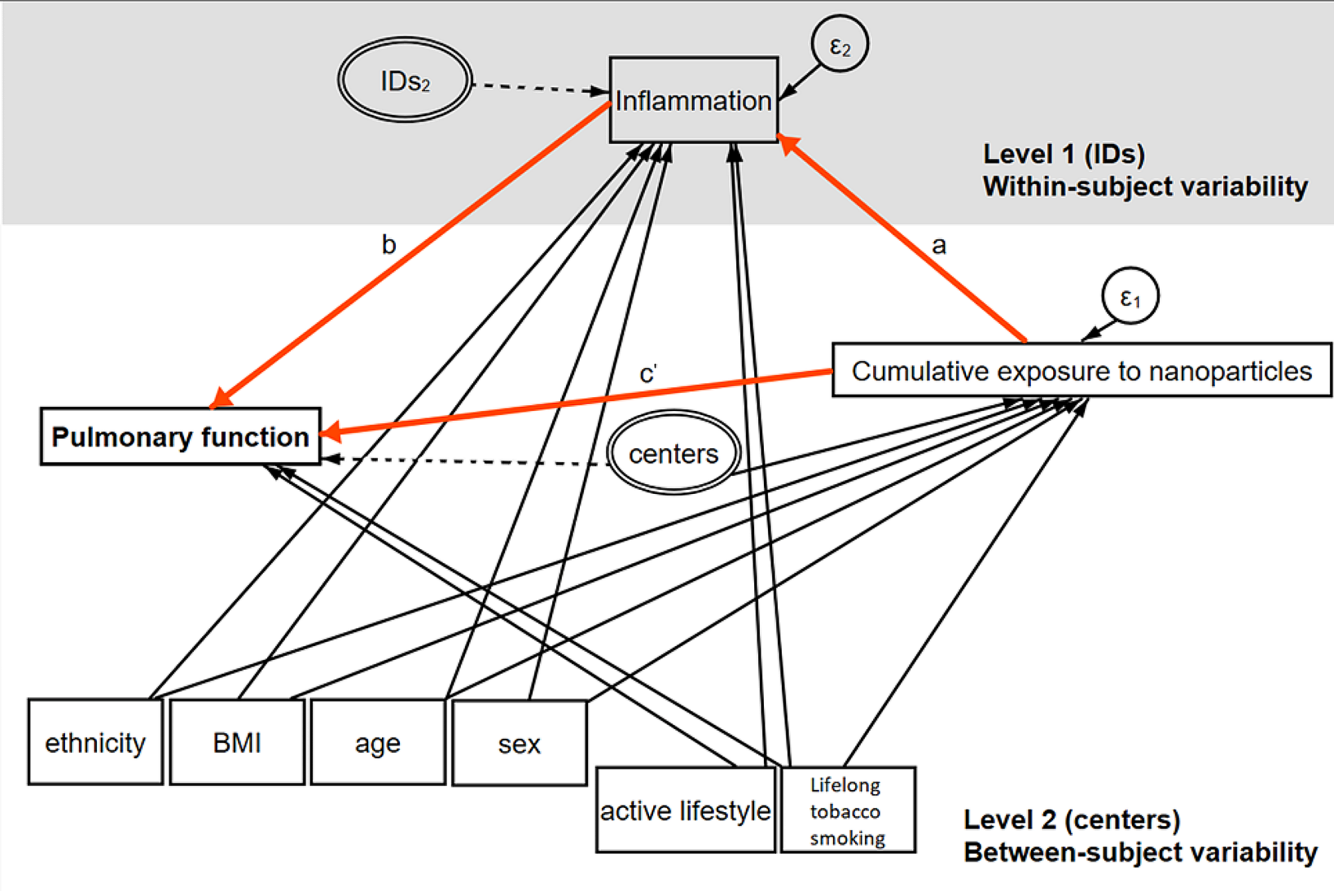
Hypothesized causal relationship between the cumulative exposure to nanomaterials and the pulmonary function. Inflammation has been measured using biomarkers (interleukins (IL): IL-10, IL-1β and TNF-α) quantified in Exhaled Breath Condensate; cumulative exposure to nanomaterials accounts for the last 10 years; pulmonary function was measured by spirometric parameter expressed as below and above the Lower Limit of Normal (LLN), i.e. below and above the fifth percentile (or a z-score < − 1.64) of the Global Lung Initiative spirometric reference equations distribution (Quanjer, 2012) of each pulmonary function parameter; centers refers to seven different recruiting centers; Active lifestyle refers to walking or cycling as commuting mode for at least 15 minutes each route or being engaged in physical activity/sport for at least twice a week and spending less than 9 hours per day being sedentary. IDs: Identification participant’s number; BMI: Body Mass Index. Path a*b: indirect effect and path c’: direct effect

Following an exploratory analysis based on logistic and linear regression models, we used generalized multilevel structural equation models (GSEM) to explore whether there was an association between cumulative exposure to nanomaterials and pulmonary function parameters, and if it could be mediated by inflammation at pulmonary level (Fig. 1). Exposure and covariates potentially acting as confounders were treated as exogenous variables, while lung function parameters and inflammatory biomarkers were set as endogenous variables, one at a time. We decided to build single-mediator models because the inflammatory biomarkers we measured in EBC are mutually influenced. For example, IL-10 is able to counteract TNF-alpha levels [38] while it inhibits IL-1beta [39]. However, because the net inflammatory balance might be of greater physiological and clinical importance than individual cytokine concentrations, an anti-pro inflammatory ratio was calculated by dividing the absolute EBC levels of IL-10 by TNF-α and then used for additional analyses.

In all GSEMs, the recruiting center and the participant’s ID were added as latent (i.e., random effect) variables accounting for the hierarchical structure of data as the participants were recruited from seven companies (level 2, between-subject variability) and inflammatory biomarkers were measured at two time points, namely at the beginning and at the end of the working week (level 1, within-subject variability). We estimated all the equations jointly and then performed a decomposition of the total effect (i.e., the association between the exposure and outcome without taking into account the mediator, the “c” pathway) into a direct effect and the indirect effect (Fig. 1, paths “c’” and “a*b”, respectively).

All results were expressed as Odds Ratios (ORs) for a given pulmonary parameter of being observed below the LLN in association with an IQR-increase of cumulative exposure to nanomaterials. We modelled two types of cumulative exposure: the particle number concentration and the LDSA. As main cumulative exposure variable we used 10-year cumulative exposures and used the 20-year one for sensitivity analyses.

The significance threshold was 0.05 and all tests were 2-sided. Mediation analysis was performed using the “gsem” command of STATA.

Statistical analyses were conducted with STATA version 18 (STATA, College Station, TX, USA) and with R version 3.6.2. Figures have been created using GraphPad Prism version 9.4.1 for Windows, GraphPad Software, San Diego, California USA, www.graphpad.com.

## Results

### Characteristics of the study population

Current analyses include 136 workers (96.5% of the initial study sample), whose characteristics are summarized in Table 1.

**Table 1:** Characteristics of the sample population

Variables	Estimate, (*n* (%))
**Participants**	136 (100%)
Age (years, mean (sd))	38.8 (10.2)
***Sex***	
Women	41 (30.2%)
Men	95 (69.8%)
BMI (mean (sd))	24.7 (3.9)
Missing data for BMI	8 (6%)
***Ethnicity***	
Caucasian	128 (94.1%)
Others	8 (5.9%)
Lifelong tobacco smoking (pack-years) (mean (sd))	4.1 (9.5)
Missing data for lifelong tobacco smoking	6 (4.4%)
***Lifestyle***	
Active	77 (56.6%)
Inactive	59 (43.4%)
Employment duration (years, mean (sd))	6.7 (7.6)
***10-year cumulative exposure to nanomaterials***	
Particle number concentration (particles/cm^3^-yrs, median (IQR))	161,692 (462,168)
LDSA (µm^2^/cm^3^-yrs, median (IQR))	305 (550)
***Pulmonary function and respiratory health***	
FEV_1_ < LLN	15 (11.6%)
FVC < LLN	8 (6.2%)
FEV_1_/FVC < LLN	10 (7.8%)
FEF_25 − 75%_ < LLN	10 (7.8%)
Missing data for all pulmonary function parameters	7 (5.1%)
Asthmatics	12 (8.8%)
COPD cases	1 (2.4%)
***Inflammatory biomarkers measured in EBC (pre-post shift average)***	
IL-10 (pg/mL, mean (sd))	1.88 (0.63)
IL-1β (pg/mL, mean (sd))	0.51 (0.20)
TNF-α (pg/mL, mean (sd))	0.19 (0.09)
IL-10/TNF-α mean (sd)	11.7 (5.4)
Missing data for all inflammatory biomarkers	7 (5.1%)

BMI: Body Mass Index, IQR: interquartile range LDSA: Lung-Deposited Surface Area, LLN: Lower Limit of Normal, i.e. respiratory parameters expressed as below and above the fifth percentile (or a z-score < − 1.64) of the Global Lung Initiative spirometric reference equations distribution (Quanjer, 2012), FEV_1_: Forced Expiratory Volume in 1 s, FVC: Forced Vital Capacity, FEF2_5 − 75%_: Forced Expiratory Fluxes at 25% and 75% of FVC, COPD: Chronic obstructive pulmonary disease; EBC: Exhaled Breath Condensate; IL: interleukin; TNF: Tumor Necrosis Factor, sd: standard deviation

### Associations between 10-year cumulative exposures to nanomaterials and pulmonary function

**Fig. 2:**
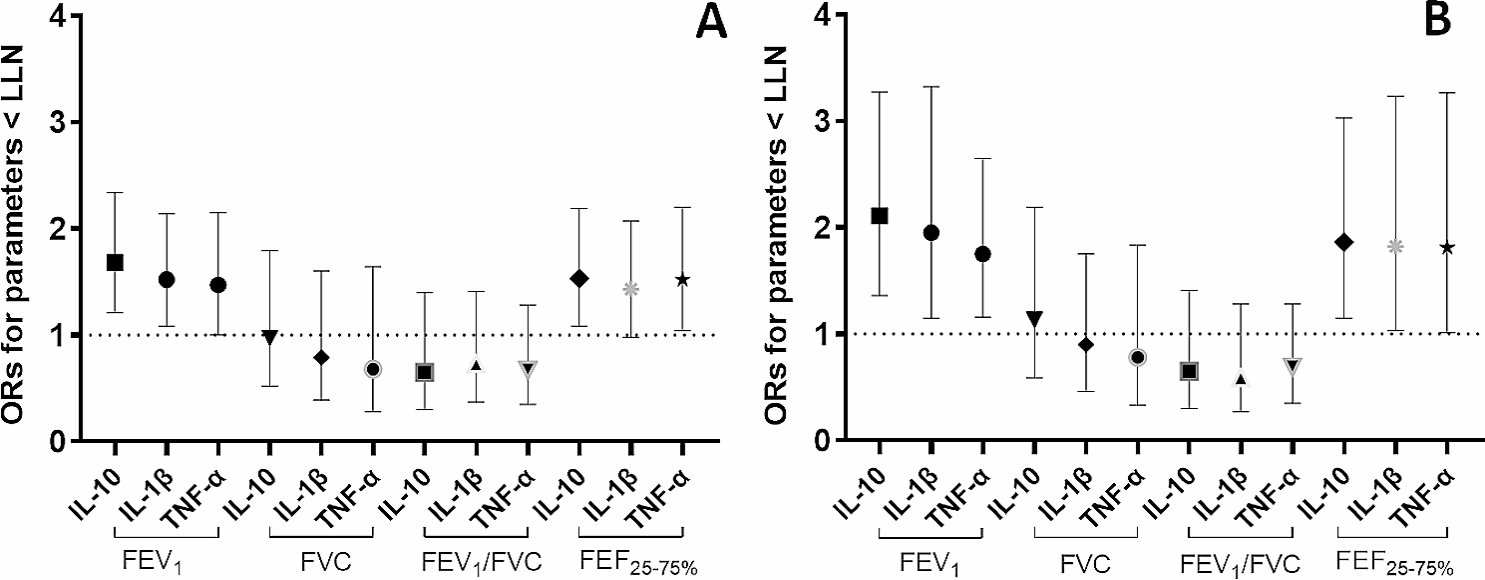
Association between 10-year cumulative exposures to nanomaterials and pulmonary function. **A**: 10-year cumulative exposure to nanomaterials expressed as particle number concentration (particles/cm^3^-years); **B**: 10-year cumulative exposure to nanomaterials expressed as Lung-Deposited Surface Area (LDSA, (µm^2^/cm^3^-years). All Odds Ratios (ORs) are derived from single-mediator Generalized Multilevel Structural Equation Models with the recruiting center and the IDs as latent variables accounting for between- and within-level variability. The ORs are calculated for an IQR-increase of cumulative exposure to nanomaterials (10 years) and are adjusted by potential confounders including active/inactive lifestyle, lifetime tobacco smoking (pack-years), sex, age, Body Mass Index (BMI) and ethnicity. The parameter-specific Lower Limit of Normal (LLN) are derived from the Global Lung Function Initiative (GLI, 2012) to express each respiratory parameter as below or above the fifth percentile (or a z-score < − 1.64) of the Global Lung Initiative spirometric reference equations distribution (Quanjer, 2012). Interleukins (ILs) and Tumor Necrosis Factor alpha are measured in exhaled breath condensate as pulmonary biomarker of inflammation

We estimated the overall associations between cumulative exposures to nanomaterials and pulmonary function, accounting for potential confounders and mediators. We found that an IQR-increase of cumulative exposure to nanomaterials, expressed as particle number concentration, was significantly associated with worse expiratory lung flows. Significantly increased odds of having a FEV_1_ below the LLN were found (OR: 1.68, 95%C.I.: 1.21–2.34, p-value = 0.002) after accounting for the individual characteristics (i.e., ethnicity, BMI, age and sex), level of inactivity, pulmonary inflammation, measured by IL-10, and lifetime tobacco smoking exposure (Fig. 2A). A slight lower but still statistically significant OR was observed when considering the other inflammatory biomarkers, the IL-1β (OR: 1.52, 95%C.I.: 1.08–2.14, p-value = 0.018) and the TNF- α (1.47, 95%C.I.: 1.002–2.150, p-value = 0.049) (Fig. 2A). As shown in Fig. 2B, stronger and statistically significant associations were detected for FEV_1_ and cumulative exposure expressed as LDSA (µm^2^/cm^3^-years) in all models (FEV_1_ < LLN, OR_IL−10_: 2.11, 95%C.I.: 1.36–3.27, p-value 0.001; OR_IL−1β_: 1.95, 95%C.I.: 1.15–3.32, p-value = 0.014; OR_TNF−α_: 1.75, 95%C.I.: 1.16–2.65, p-value = 0.008).

The odds of observing abnormal FEF_25 − 75%_ values increased by around 53% in association with the cumulative exposure expressed as particle number concentration when accounting for the effect of IL-10 and TNF-α (OR: 1.53, 95% I.C.: 1.08–2.19, p-value = 0.018 and OR: 1.52, 95% I.C.: 1.04–2.20, p-value = 0.029, respectively). For IL-1β the estimated odds were similar yet non-significant (OR: 1.43, 95% I.C.: 0.98–2.07, p-value = 0.063) (Fig. 2A). Stronger associations were confirmed for the exposure expressed as LDSA (FEF _25−75%_ < LLN, OR_IL−10_: 1.86, 95%C.I.: 1.15–3.03, p-value = 0.012; OR_IL−1β_: 1.82, 95%C.I.: 1.03–3.23, p-value = 0.041; OR_TNF−α_: 1.81, 95%C.I.: 1.01–3.26, p-value = 0.048) (Fig. 2B). Conversely, no associations between cumulative exposure to nanomaterials and FVC, nor FEV_1_/FVC ratio were observed (Fig. 2A and B).

Independently of cumulative exposure to nanomaterials expressed as particle number concentration, results suggest that subjects engaging an active lifestyle seem protected against the risk of having the FEV_1_ below the LLN (OR_IL−10_: 0.27, 95%C.I.: 0.10–0.69, p-value = 0.006; OR_IL−1β_: 0.32, 95%C.I.: 0.13–0.79, p-value = 0.014; OR_TNF−α_: 0.31, 95%C.I.: 0.13–0.77, p-value = 0.012) (Table S2). Similar results were found against the risk of having the FEV_1_ below the LLN in the model with cumulative exposure expressed as LDSA (FEV_1_ < LLN, OR_IL−10_: 0.27, 95%C.I.: 0.11–0.70, p-value = 0.007; OR_IL−1β_: 0.32, 95%C.I.: 0.13–0.80, p-value = 0.014; OR_TNF−α_: 0.32, 95%C.I.: 0.13–0.80, p-value = 0.014) (Table S3). An analogous trend was observed for the association between an active lifestyle and FEV_1_/FVC, but not for the FVC and FEF_25 − 75%,_ in the models with cumulative exposure to nanomaterials expressed as particle number concentration (Table S2). The same findings, with a percentage variation between estimates ranging from 0 to 9.5%, were observed when expressing the exposure as LDSA (Table S3).

Additionally, lifetime smoking exposure (i.e., pack-years) was associated with an increased risk of worse respiratory health in terms of both FEV_1_ (OR_IL−10_: 1.05, 95%C.I.: 1.01–1.08, per pack-years, p-value = 0.019; OR_IL−1β_: 1.040, 95%C.I.: 1.004–1.080, per pack-years, p-value = 0.028; OR_TNF−α_: 1.04, 95%C.I.: 1.001–1.070, per pack-years, p-value = 0.046) and FVC (OR_IL−10_: 1.05, 95%C.I.: 1.01–1.09,, per pack-years, p-value = 0.010; OR_IL−1β_: 1.05, 95%C.I.: 1.01–1.10, per pack-years, p-value = 0.013; OR_TNF−α_: 1.05, 95%C.I.: 1.01–1.10, per pack-years, p-value = 0.023) but not FEV_1_/FVC and FEF_25 − 75%_ (Table S2). Similar results were found in the models with LDSA as exposure variable (Table S3).

Furthermore, a dose-response relationship was found between pulmonary inflammation and cumulative exposure to nanomaterials. As reported by Table 2, both anti-inflammatory (i.e., IL-10) and pro-inflammatory (i.e., TNF-α and IL-1β) biomarkers were significantly higher (*p* < 0.0001) in association with 10-year cumulative exposure to nanomaterials.

**Table 2:** Levels of pulmonary inflammation in association with 10-year cumulative exposure to nanomaterials

Inflammatory biomarkers (pg/mL)	β (95% CI)	*p*-value
**IL-10** ^**a, b,c, d**^	0.31 (0.16–0.45)	< 0.0001
**IL-10** ^**e, f,g, h**^	0.37 (0.23–0.52)	< 0.0001
**IL-1β** ^**a, b,c, d**^	0.34 (0.20–0.47)	< 0.0001
**IL-1β** ^**e, f,g, h**^	0.46 (0.29–0.62)	< 0.0001
**TNF-α** ^**a, b,c, d**^	0.48 (0.31–0.65)	< 0.0001
**TNF-α** ^**e, f,g, h**^	0.42 (0.25–0.58)	< 0.0001

^a, b,c, d^ models including cumulative exposure expressed as particle number concentration and lung function parameters, namely FEV_1_, FVC, FEV_1_/FVC and FEF_25 − 75%_, one at a time; ^e f g h^ models including cumulative exposure expressed as Lung-Deposited Surface Area (LDSA) and lung function parameters, namely FEV_1_, FVC, FEV_1_/FVC and FEF_25 − 75%_, one at a time

All estimates are derived from single-mediator Generalized Multilevel Structural Equation Models with the recruiting center and the IDs as latent variables accounting for between- and within-level variability. The ORs are calculated for an IQR-increase of cumulative exposure to nanomaterials (10 years) and are adjusted by potential confounders including active/inactive lifestyle, lifetime tobacco smoking (pack-years), sex, age, Body Mass Index (BMI) and ethnicity. The parameter-specific Lower Limit of Normal (LLN) are derived from the Global Lung Function Initiative (GLI, 2012) to express each respiratory parameter as below or above the fifth percentile (or a z-score < − 1.64) of the Global Lung Initiative spirometric reference equations distribution (Quanjer, 2012). Interleukins (ILs) and Tumor Necrosis Factor alpha are measured in exhaled breath condensate as pulmonary biomarker of inflammation

### Mediation effect of pulmonary biomarkers of inflammation: single-biomarker mediation analysis

After the decomposition of the total effect (i.e., the association between the exposure and outcome without accounting for the mediator) into a direct and indirect effect (pathways “c’” and “a*b”. in Fig. 1, respectively), we generally did not observe any mediation (Table 3), with the only exception of the association between cumulative exposure to nanomaterials and FEV_1_/FVC parameter. In particular, a statistically significant indirect effect on FEV_1_/FVC via TNF-α was observed when accounting for both cumulative exposures, namely particle number concentration and LDSA (OR = 1.21, 95%C.I.:1.01–1.44 and 1.26, 95%C.I.:1.01–1.56, respectively) (Table 3). In addition, IL-10 seemed to fully mediate the association between cumulative exposure to nanomaterials and FEV_1_/FVC (OR = 0.78 95%C.I.:0.59–0.97 and OR = 0.66 95%C.I.: 0.41–0.91 for particle number concentration and LDSA, respectively) but in the opposite direction as compared to the mediating effect exerted by the TNF-α (Table 3).

**Table 3:** Mediation effect via pulmonary inflammatory mediators

Lung function parameters	10-year cumulative exposure as particle number concentration (particles/cm^3^-yrs)			10-year cumulative exposure as LDSA (µm^2^/cm^3^-yrs)		
	Via IL-10 OR (95% C.I.)	Via IL-1β OR (95% C.I.)	Via TNF-α OR (95% C.I.)	Via IL-10 OR (95% C.I.)	Via IL-1β OR (95% C.I.)	Via TNF-α OR (95% C.I.)
FEV_1_						
Indirect effect	0.93 (0.79–1.06)	1.06 (0.87–1.26)	1.00 (0.80–1.18)	0.84 (0.63–1.04)	1.15 (0.80–1.49)	1.01 (0.81–1.22)
Direct effect	**1.68 (1.21–2.34)**	**1.52 (1.08–2.14)**	1.470 (1.002–2.150)	**2.11 (1.36–3.27)**	**1.95 (1.15–3.32)**	**1.75 (1.16–2.65)**
Total effect	1.56 (1.07–2.05)	1.61 (1.08–2.14)	1.46 (0.84–2.08)	**1.76 (1.20–2.59)**	**2.24 (1.19–4.1)**	**1.77 (1.09–2.46)**
**FVC**						
Indirect effect	0.94 (0.76–1.13)	1.1 (0.85–1.35)	0.94 (0.69–1.18)	0.89 (0.62–1.16)	1.10 (0.78–1.43)	0.94 (0.66–1.22)
Direct effect	0.97 (0.52–1.79)	0.79 (0.39–1.60)	0.68 (0.28–1.64)	1.13 (0.59–2.19)	0.90 (0.46–1.75)	0.78 (0.33–1.83)
Total effect	0.91 (0.39–1.43)	0.87 (0.30–1.43)	0.63 (0.03–1.24)	1.01 (0.42–1.59)	0.99 (0.39–1.59)	0.73 (0.06–1.42)
**FEV**_**1**_ **/FVC**						
Indirect effect	**0.78 (0.59–0.97)**	1.09 (0.88–1.30)	**1.21 (1.01–1.44)**	**0.66 (0.41–0.91)**	0.98 (0.64–1.32)	**1.26 (1.01–1.56)**
Direct effect	0.65 (0.30–1.40)	0.73 (0.37–1.41)	0.67 (0.35–1.28)	0.65 (0.30–1.41)	0.59 (0.27–1.28)	0.69 (0.35–1.28)
Total effect	0.51 (0.23–1.10)	0.80 (0.30–1.28)	0.81 (0.29–1.32)	**0.43 (0.07–0.79)**	0.57 (0.06–1.08)	0.84 (0.30–1.38)
**FEF**_**25 − 75%**_						
Indirect effect	0.89 (0.74–1.10)	1.00 (0.80–1.19)	0.92 (0.75–1.09)	0.80 (0.58–1.03)	1.10 (0.73–1.47)	0.91 (0.66–1.16)
Direct effect	**1.53 (1.08–2.19)**	1.43 (0.98–2.07)	**1.52 (1-04-2.20)**	**1.86 (1.15–3.03)**	**1.82 (1.03–3.23)**	**1.81 (1.01–3.26)**
Total effect	1.37 (0.90–1.85)	**1.42 (1.00-2.03)**	1.40 (0.91–1.88)	1.50 (0.83–2.17)	**2.00 (1.01–3.98)**	1.65 (0.59–2.71)

All Odds Ratios (ORs) are derived from the decomposition into direct and indirect effects estimated by single-mediator Generalized Multilevel Structural Equation Models with the recruiting center and the IDs as latent variables accounting for between- and within-level variability. The ORs are calculated for an IQR-increase of cumulative exposure to nanomaterials (10 years) and are adjusted by potential confounders including active/inactive lifestyle, lifetime tobacco smoking (pack-years), sex, age, Body Mass Index (BMI) and ethnicity. The parameter-specific Lower Limit of Normal (LLN) are derived from the Global Lung Function Initiative (GLI, 2012) to express each respiratory parameter as below or above the fifth percentile (or a z-score < − 1.64) of the Global Lung Initiative spirometric reference equations distribution (Quanjer, 2012). Interleukins (ILs) and Tumor Necrosis Factor alpha are measured in exhaled breath condensate as pulmonary biomarker of inflammation

### Associations between 10-year cumulative exposures to nanomaterials and their effect on the anti-pro inflammatory ratio

As additional analysis, both main models and mediation analysis have been mirrored using an anti-pro inflammatory ratio, namely IL-10/TNF-α, instead of a single inflammatory mediator (Table S4 and Table 4). We generally observed that, as already reported in the main analyses, only FEV_1_ and FEF_25 − 75%_ were associated with cumulative exposure to nanomaterials. However, many associations were not statistically significant anymore (Table S4). Interestingly, for each IQR-increase of 10-year cumulative exposures there was a decrease of the anti-pro inflammatory ratio, equal to -1.23 (95%CI: -2.14; -0.32; *p* = 0.008) and − 1.28 (95%CI: -2.35; -0.20; *p* = 0.020), when considering the exposure expressed as particle number concentration or LDSA, respectively (Table S4). Table 4 reports the decomposition of the total effect into direct and indirect effects of the cumulative exposure to nanomaterials on pulmonary function via the anti-pro inflammatory ratio as a potential mediator. Also in this case, only the association with FEV_1_/FVC showed a trend of a full mediation via the anti-pro inflammatory ratio.

**Table 4:** Indirect effect of cumulative exposures to nanomaterials on lung function via the anti-pro inflammatory ratio

Lung function parameters	10-year cumulative exposure as particle number concentration (particles/cm^3^-yrs)	10-year cumulative exposure as LDSA (µm^2^/cm^3^-yrs)
	Via IL-10/TNF-α OR (95% C.I.)	Via IL-10/TNF-α OR (95% C.I.)
FEV_1_		
Indirect effect	1.06 (0.93–1.19)	1.07 (0.93–1.21)
Direct effect	**1.46 (1.02–2.15)**	**1.72 (1.17–2.53)**
Total effect	**1.57 (1.06–2.34)**	**1.85 (1.24–2.76)**
**FVC**		
Indirect effect	1.06 (0.90–1.23)	1.07 (0.90–1.24)
Direct effect	0.74 (0.33–1.71)	0.84 (0.37–1.91)
Total effect	0.80 (0.10–1.49)	0.90 (0.13–1.66)
**FEV**_**1**_ **/FVC**		
Indirect effect	**1.17 (1.00-1.37)**	**1.17 (1.00-1.39)**
Direct effect	0.76 (0.42–1.38)	0.76 (0.42–1.41)
Total effect	0.89 (0.34–1.43)	0.90 (0.32–1.47)
**FEF**_**25 − 75%**_		
Indirect effect	1.00 (0.89–1.11)	1.01 (0.89–1.13)
Direct effect	**1.43 (1.00-2.05)**	**1.81 (1.02–3.20)**
Total effect	**1.420 (1.003–1.920)**	**1.82 (1.02–2.91)**

All Odds Ratios (ORs) are derived from the decomposition into direct and indirect effects estimated by single-mediator Generalized Multilevel Structural Equation Models with the recruiting center and the IDs as latent variables accounting for between- and within-level variability. The ORs are calculated for an IQR-increase of cumulative exposure to nanomaterials (10 years) and are adjusted by potential confounders including active/inactive lifestyle, lifetime tobacco smoking (pack-years), sex, age, Body Mass Index (BMI) and ethnicity. The parameter-specific Lower Limit of Normal (LLN) are derived from the Global Lung Function Initiative (GLI, 2012) to express each respiratory parameter as below or above the fifth percentile (or a z-score < − 1.64) of the Global Lung Initiative spirometric reference equations distribution (Quanjer, 2012). The anti-pro inflammatory ratio has been calculated by dividing IL-10 levels by TNF-α, both quantified in exhaled breath condensate as pulmonary biomarker of inflammation

### Associations between 20-year cumulative exposures to nanomaterials and pulmonary function

**Fig. 3:**
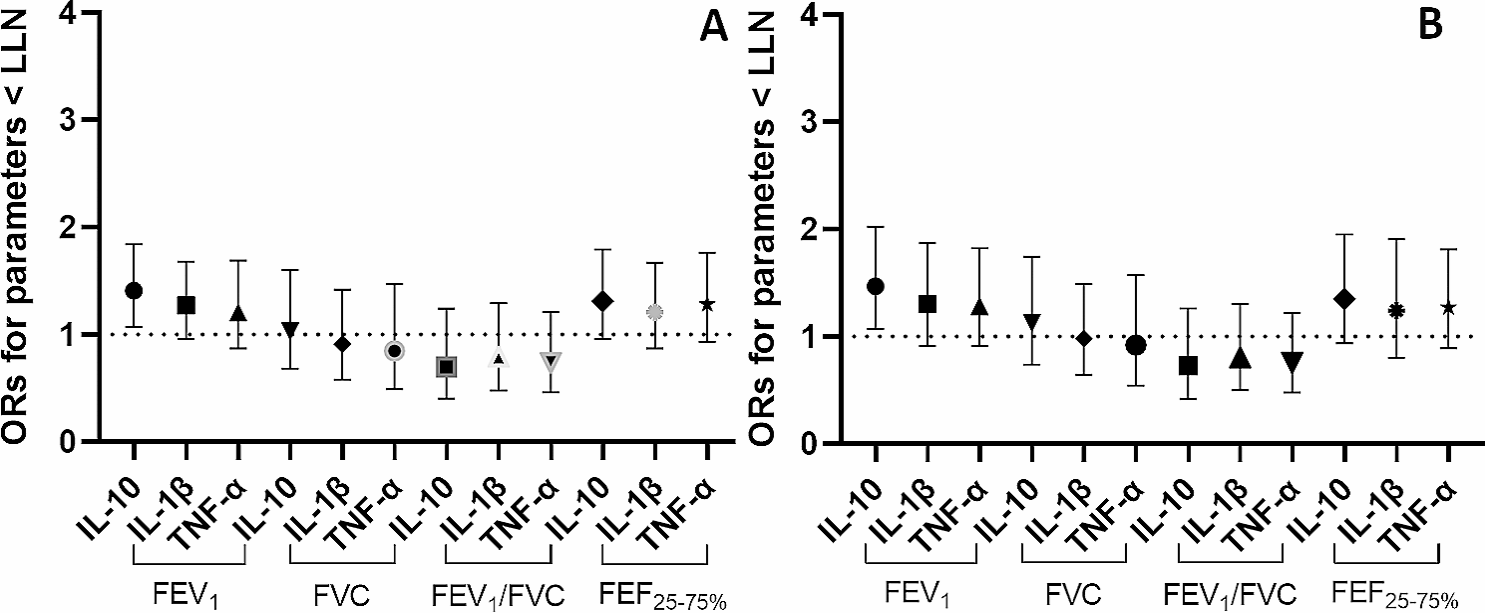
Association between 20-year cumulative exposures to nanomaterials and pulmonary function. **A**: 20-year cumulative exposure to nanomaterials expressed as particle number concentration (particles/cm^3^-years); **B**: 20-year cumulative exposure to nanomaterials expressed as Lung-Deposited Surface Area (LDSA, (µm^2^/cm^3^-years). All Odds Ratios (ORs) are derived from single-mediator Generalized Multilevel Structural Equation Models with the recruiting center and the IDs as latent variables accounting for between- and within-level variability. The ORs are calculated for an IQR-increase of cumulative exposure to nanomaterials (20 years) and are adjusted by potential confounders including active/inactive lifestyle, lifetime tobacco smoking (pack-years), sex, age, Body Mass Index (BMI) and ethnicity. The parameter-specific Lower Limit of Normal (LLN) are derived from the Global Lung Function Initiative (GLI, 2012) to express each respiratory parameter as below or above the fifth percentile (or a z-score < − 1.64) of the Global Lung Initiative spirometric reference equations distribution (Quanjer, 2012). Interleukins (ILs) and Tumor Necrosis Factor alpha are measured in exhaled breath condensate as pulmonary biomarker of inflammation

Finally, in the sensitivity analyses, the same approach was used to test the association between 20-year cumulative exposures and pulmonary function (Fig. 3A and B). Although most of the associations lost statistical significance, a similar trend was confirmed, especially when accounting for cumulative exposure expressed as particle number concentration.

More in details, significantly increased odds of having a FEV_1_ below the LLN were found in association with both the particle number concentration and LDSA (OR: 1.41, 95%C.I.: 1.07–1.86, p-value = 0.015; OR: 1.47, 95%C.I.: 1.07–2.02, p-value = 0.017) after accounting for pulmonary inflammation, measured by IL-10, and confounders (Fig. 3A and B). The associations via other inflammatory biomarkers showed similar trends but lost significance when accounting for the particle number concentration (FEV_1_ < LLN: OR_IL−1β_: 1.27, 95%C.I.: 0.96–1.68, p-value = 0.093; OR_TNF−α_: 1.21, 95%C.I.: 0.87–1.69, p-value = 0.255) and LDSA (FEV_1_ < LLN: OR_IL−1β_: 1.28, 95%C.I.: 0.91–1.82, p-value = 0.160; OR_TNF−α_: 1.30, 95%C.I.: 0.91–1.87, p-value = 0.155) (Fig. 3A and B). The odds of observing abnormal FEF_25 − 75%_ values showed a trend in association with the cumulative exposure expressed as particle number concentration when accounting for the effect of IL-10 (OR: 1.31, 95% I.C.: 0.96–1.79, p-value = 0.085). For IL-1β and TNFα the estimated odds were similar yet non-significant (OR: 1.21, 95% I.C.: 0.88–1.67, p-value = 0.253 and OR: 1.28, 95% I.C.: 0.93–1.76, p-value = 0.138, respectively) (Fig. 3A). Similar results were observed for the exposure expressed as LDSA (FEF _25−75%_ < LLN: OR_IL−10_: 1.35, 95%C.I.: 0.94–1.95, p-value = 0.103; OR_IL−1β_: 1.24, 95%C.I.: 0.80–1.91, p-value = 0.333; OR_TNF−α_: 1.27, 95%C.I.: 0.89–1.81, p-value = 0.193) (Fig. 3B).

Results on the mediation effect via IL-10 (OR: 0.80, 95%C.I.: 0.64–0.97, p-value = 0.037 and OR: 0.76, 95%C.I.: 0.59–0.98, p-value = 0.036 for particle number concentration and LDSA, respectively) and TNF-α (OR: 1.150, 95%C.I.: 1.004–1.310, p-value = 0.043 and OR: 1.150, 95%C.I.: 1.001–1.312, p-value = 0.049 for particle number concentration and LDSA, respectively) hold.

## Discussion

This study focused on the association between cumulative exposure to nanomaterials and pulmonary function in the NanoExplore cohort. We observed that for each IQR-increase of 10-year cumulative exposure to nanomaterials there were worse FEV_1_ and FEF_25-75%_. This might indicate that both the large and small airway components are likely to be involved. When we expressed the cumulative exposure as particle number concentration the effect was slightly smaller as compared to the one observed in association with exposure expressed as LDSA, while more imprecise (i.e., larger CIs). Noteworthy, since the LDSA metric reflects the concentration of particles reaching the alveolar region of the human lungs, its strongest associations with health-related effects meet the biological plausibility criterion and reinforce the biological significance of our results. Given the interaction between respiration and pulmonary circulation occurring in the alveolar region of the lungs, the strongest associations with LDSA suggest the possibility of broader effects, exerted by nanomaterials on human health. We further explored the hypothesis that the observed associations could be mediated by airway inflammation, assessed by three inflammatory biomarkers quantified in the EBC of workers, and the derived anti-pro inflammatory ratio. Although explorative, the mediation analysis suggested that TNF-α may have a full mediating role (i.e., both c and c’ coefficients are non-significant) in the association between cumulative exposure to nanomaterials and the risk of having FEV_1_/FVC below the LLN, but not in that with other pulmonary function parameters. The mediation effect on the same parameter was confirmed for IL-10 levels and, marginally, for the anti-pro inflammatory ratio. Overall, our findings suggest an effect of cumulative exposure to nanomaterials on respiratory function, which might be consistent with early signs of airflow obstruction. Although we did not observe any direct effect of exposure on FEV_1_/FVC, the mediation analysis provided newsworthy hints on an indirect effect, i.e., the increased risk of abnormal FEV_1_/FVC via the increase of pulmonary TNF-α or via the decrease of the IL-10/TNF-α ratio.

On the sidelines but not less important, workers reporting an active lifestyle seem to be strongly protected against the risk of having a worse pulmonary function, with an effect on FEV_1_, FEV_1_/FVC, but not on FVC and FEF_25-75%_. As expected, lifelong tobacco smoking exposure contributed to poorer respiratory health, exerting its detrimental effect on FEV_1_ and FVC parameters.

Although existing literature on this topic is scarce, especially on measured cumulative exposure, our findings align with previous reports indicating that the exposure to various types of nanomaterials is generally associated with respiratory impairments. In a six-month follow-up study involving 124 nanomaterial-handling workers from 14 manufacturing plants in Taiwan [12], the authors reported significantly stronger decreases in pulmonary function parameters (Maximal mid-expiratory flow, peak expiratory flow rate and forced expiratory flow at 25%) in exposed workers as compared to the unexposed controls. In their pre-post shift investigation, Pelclova et al. [8] observed a significant post-shift decline of FEV_1_ and FEV_1_/FVC in 20 workers from the nanocomposite sector, whose duration of employment was also positively associated with post-shift decline in pulmonary function parameters. Cao and colleagues [16] observed a worse lung function in 58 Chinese workers chronically exposed to nanoscale carbon black and demonstrated that airway wall thickening is a major pathophysiological mechanism via which the exposure to carbon black affects pulmonary function. Findings from cross-sectional studies align with the aforementioned ones. Zhang and colleagues [11] reported that occupational exposure to nanoscale carbon black particles could be responsible for the pulmonary function reduction and inflammatory mediator secretion. The authors observed lower FEV_1_, FEV_1_/FVC, peak expiratory flow and maximal mid-expiratory flow in 81 Chinese workers exposed to carbon black nanoparticles as compared to controls. In another cross-sectional study, Zhao and colleagues [9] reported significantly lower pulmonary parameters in 83 Chinese workers exposed to nano-TiO_2_, as compared with a control group. On the contrary, no significant effect of occupational exposure to printer emissions was observed on pulmonary function among 53 Chinese workers although, the average value of each parameter tended to be lower, when compared with a group of unexposed controls [15].

Existing literature from population-based studies is only partly comparable because it mainly focused on different exposures (e.g., mineral or biological dust), generally assessed using a job-exposure matrix, and not specifically focusing on the nanocomposite sector. For example, people occupationally exposed to biological dust, mineral dust and metals showed an accelerated pulmonary function decline per 25-intensity-years exposure, comparable in magnitude with that associated with long-term smoking [40]. Occupational exposure-associated risk of obstructive diseases such as COPD was observed mainly in males and ages ≥ 40 years from the Swiss working adult population exposed to biological dusts, mineral dusts, gases/fumes, and remained elevated when restricted to non-smokers [41]. In French subway workers a dose-response relationship with cumulative exposure to subway particles was observed for COPD diagnosis and self-reported asthma. The latter was also associated with atopic sensitization and nitrite concentration measured in EBC of subway workers [27]. A systematic review on the available longitudinal data on lung function decline and respiratory symptoms in welders suggests that welding may be associated with an accelerated decline in lung function, particularly in combination with smoking [42]. Another recent systematic review on current and cumulative occupational exposure and lung function decline highlighted that exposure to mineral dusts and metals was not significantly associated with FEV_1_ decline [43].

Evidence from experimental animal studies suggests that the inhalation of nanomaterials is associated with a plethora of biological mechanisms, such as oxidative stress and inflammation, and health outcomes including pulmonary fibrosis, granuloma, lung cancer, mesothelioma-like effects, cardiovascular effects, and pleural plaque formation [12]. In humans, given the paucity of epidemiological studies on the effect of exposure to nanomaterials, the biological plausibility underlying their toxic effects can be mostly inferred from in vivo or in vitro studies or, alternatively, from epidemiological studies on ambient ultrafine particles (UFPs). However, although UFPs and nanoparticles share a common feature of size (one dimension < 100 nm), analogies on their toxicological effects should be taken with caution as they differ in many other physicochemical properties such as composition, surface coatings and reactivity [9]. Additionally, existing literature on UPF-related effects can provide limited supporting evidence as, in many cases, mixed results have emerged. For example, several studies reported that exposure to black carbon and PM_2.5_ led to increased rate of decline of lung function parameters in adults [44, 45], while other authors did not observe any significant associations [46].

As already mentioned, oxidative stress and inflammation are the two main mechanisms by which nanomaterials are supposed to exert their toxic effects on respiratory and general health. Exposure to nanomaterials is able to induce pulmonary oxidative stress and inflammation [47], and this is in line with our results on the observed dose-response relationship between cumulative exposures to nanomaterials and pulmonary levels of inflammation. Moreover, the mediation analysis corroborates this result suggesting that IL-10, TNF-α, and their ratio fully mediate the association between the cumulative exposure to nanomaterials and FEV_1_/FVC. Exposure to nanomaterials and the derived inflammation may influence the activation of muscarinic receptors that control the smooth muscle tone [48] which can affect the airway tightening, thus the lung function decline. Similar mechanisms were also observed in rat bronchi segments under the effect of an experimental exposure to PM_2.5_ [49]. These mechanisms are consistent with the obstructive signs observed in the present study, and may partly explain them from a biological plausibility perspective.

In our study, the mediation analysis served as a preliminary investigation on the contribution of local inflammatory levels in the process that could results in pulmonary impairments, especially if prolonged over time and in specific groups at higher risk (e.g., more exposed workers). Although preliminary, our findings suggest that the higher the cumulative exposure levels, the higher concentration of inflammatory cytokines are detected in the lung. As per TNF-α, we observed that cumulative exposure to nanomaterials over 10 and 20 years is associated with increased pulmonary inflammation, which in turn may imply worse respiratory health in terms of FEV_1_/FVC. This is in line with the biological meaning of this pro-inflammatory biomarker, as it plays a crucial role in many inflammatory respiratory diseases, especially those characterized by obstruction such as chronic bronchitis, COPD and asthma [50]. On the contrary, the IL-10, a T-helper cell type 2 cytokine, has a broad spectrum of anti-inflammatory actions [51] and this might partly support our results that suggest that nanomaterial-induced IL-10 in the lung can be associated with a risk reduction in terms of FEV_1_/FVC. Noteworthy, the analysis of the anti-pro inflammatory ratio not only confirmed the mediating role of the pulmonary inflammation levels but also highlighted that the dysregulation between pro- and anti-inflammatory might be accentuated, in favor of pro-inflammatory mediators, by a long-lasting exposure to nanomaterials. Perturbations on the net inflammatory balance might be of greater physiological and clinical importance than individual cytokine concentration. Previous clinical studies highlighted the importance of cytokine balance in the lungs of patients suffering from Acute Respiratory Distress Syndrome (ARDS) [52], community-acquired pneumonia [53], and COPD [54].

Our findings must be interpreted in the context of certain limitations. First, the study design limited the possibility to assess the temporal criterion that should exist between exposure, alterations in inflammatory biomarkers, and changes in respiratory function parameters, especially when analyzing the causal pathway. Indeed, the collection of biological samples, pre and post shift, was performed almost simultaneously with the execution of respiratory function tests. Nevertheless, it is possible to hypothesize that, under similar exposure conditions over time, the observed inflammatory markers could serve as a reliable indicator of the local inflammation pattern during the period of work for the employees involved in the analysis. Second, cumulative exposure was derived by multiplying the employment duration by the particle number concentration assessed during field campaigns assuming that it might have been representative of the last year of exposure. Although we are aware of the strong assumptions behind this choice, which are challenging to verify for the time being, we operated in a conservative way. Indeed, it is likely that occupational exposures tend to generally decrease in compliance with the provision of health and safety requirements, thus we might have actually underestimated the exposure levels. Third, the sample size could be inadequate to properly investigate the association between cumulative exposure to nanomaterials and respiratory health effects. Indeed, as declared in the study protocol [5], pulmonary function parameters were considered as secondary outcomes and sample size calculations were based on the early biological effects instead. Fourth, in this analysis we exploited data collected at the cohort set-up (i.e., cross-sectionally) and retrospectively estimated the nanomaterials exposure 10 and 20 years backward to ensure the temporality condition. Indeed, it is only by reassessing the analyzed relationships, using prospectively collected data over the next 10–20 years, that the confirmation of these results would become possible. Although we detected significant associations between the exposure and outcomes considered in the main analysis, the possible sample size underestimation and a too long exposure duration (i.e., 20 years) might have contributed to the loss of significance levels in the sensitivity analyses. Noteworthy, the relatively small sample size of this cohort corresponds to one of the largest epidemiological studies of workers exposed to nanomaterials in the world. Fifth, it was not possible to include in the model the variable related to the presence of obstructive respiratory pathologies, such as asthma and COPD, due to their low prevalence in the sample population (< 9% and 0.7%, respectively). This phenomenon, especially regarding COPD, could also be attributed to the “healthy worker effect” [55], that might have contributed to attenuate the estimates referred to the longer cumulative exposure (i.e., 20 years). The latter, could be also have been an exaggerated case scenario, as the average employment duration in our epidemiological sample was closer to 10 years (mean ± SD: 8.4 ± 7.7 years) than 20 years. In addition, we could not test the association between the exposure to different types of nanomaterials and health effects because the sample size was too limited to allow a stratified analysis. Noteworthy, workers were exposed to complex mixtures rather than a few nanomaterials at a time and, although the nanomaterials used by the companies showed a certain degree of variation, they partly overlapped for some workers from different companies. However, is advisable for future studies to fill this gap. Finally, as several hypotheses were tested with different respiratory parameters, different inflammatory biomarkers and different exposure estimates, the multiplicity might be an issue.

While the validity of our findings requires further confirmation by prospective studies that can capture the cause-effect relationship, this study has also several strengths. First, the NanoExplore project is based on a harmonized protocol [5] that has been rigorously applied across seven centers located in three countries, which enables a certain degree of results generalization within similar nanomaterials productive patterns. Secondly, given the complexity of exposures to different nanomaterials in the workplace, human biomonitoring is a key approach able to integrate different exposure pathways to assess the early biological effects. Additionally, we opted for a non-invasively collected biological matrix, the EBC, that has been employed to quantify local inflammation accounting for intra and inter-subject variability among workers. Indeed, the quantification of inflammatory biomarkers in EBC is supposed to be able to reflect the pulmonary microenvironment and eventual variations of inflammatory levels during the working week and in association with cumulative exposure to nanomaterials.

## Conclusions

In conclusion, the cumulative exposure to nanomaterials was associated with worse FEV_1_ and FEF_25 − 75%_ in the largest existing cohort of workers from the nanocomposite sector, plus a group of unexposed workers. The observed associations were independent of lifelong tobacco smoking, ethnicity, age, sex, BMI and physical activity habits. Further, the anti-pro inflammatory ratio, measured in EBC, was reduced in association with cumulative exposure to nanomaterials and seemed to fully mediate, as already observed for IL-10 and TNF-α, an indirect effect on FEV_1_/FVC.

In light of these findings, safeguarding the respiratory health of workers exposed to nanomaterials should be considered of primary importance. The observed association between cumulative exposure to nanomaterials and worse pulmonary function underscores the importance of implementing adequate protective measures. Altogether, this study contributed to improve existing evidence on the effects of exposure to nanomaterials, which should be a cause for concern from Public and Occupational health perspectives. The mitigation of harmful exposures may ensure that workers can continue to contribute productively to their workplaces while preserving their respiratory health over time. Further prospective cohort studies are needed to thoroughly explore the cause-effect relationship between alterations in inflammatory mediators and changes in pulmonary function.

### Electronic supplementary material

Below is the link to the electronic supplementary material.

Supplementary Material 1

## Data Availability

No datasets were generated or analysed during the current study.
