# Supplementary material for: Worse pulmonary function in association with cumulative exposure to nanomaterials. Hints of a mediation effect via pulmonary inflammation
Source: Part Fibre Toxicol. 2024 Jun 28;21:28. doi: 10.1186/s12989-024-00589-3 (PMC11212158; doi:10.1186/s12989-024-00589-3)
Supplement: Supplementary file 1 — Supplementary Material 1 [file 12989_2024_589_MOESM1_ESM.docx]

**Supplementary material**

**Worse pulmonary function in association with cumulative exposure to nanomaterials. Hints of a mediation effect via pulmonary inflammation.**

Giulia Squillacioti^1^, Thomas Charreau^2^, Pascal Wild^2^, Valeria Bellisario^1^, Federica Ghelli^1^, Roberto Bono^1^, Enrico Bergamaschi^1, 3^, Giacomo Garzaro^1, 3 *^, Irina Guseva Canu^4*^

^1^ Department of Public Health and Pediatrics, University of Turin, Via Santena 5 bis, 10126 Torino, Italy;

^2^ Department of Occupational and Environmental Health, Center for Primary Care and Public Health (Unisanté), University of Lausanne, 1066 Epalinges, Lausanne, Switzerland;

^3^ Città della Salute e della Scienza di Torino, University Hospital, Via Zuretti 29, 10126 Turin, Italy;

^4^ Department of Occupational and Environmental Health, Center for Primary Care and Public Health (Unisanté), University of Lausanne, 1066 Epalinges, Lausanne, Switzerland.

Corresponding author: Prof. Roberto Bono, roberto.bono@unito.it; Department of Public Health and Pediatrics, University of Turin, Via Santena 5 bis, 10126 Torino, Italy

* These two authors contributed equally

**Figure S1.** Diagram of participants selection

**
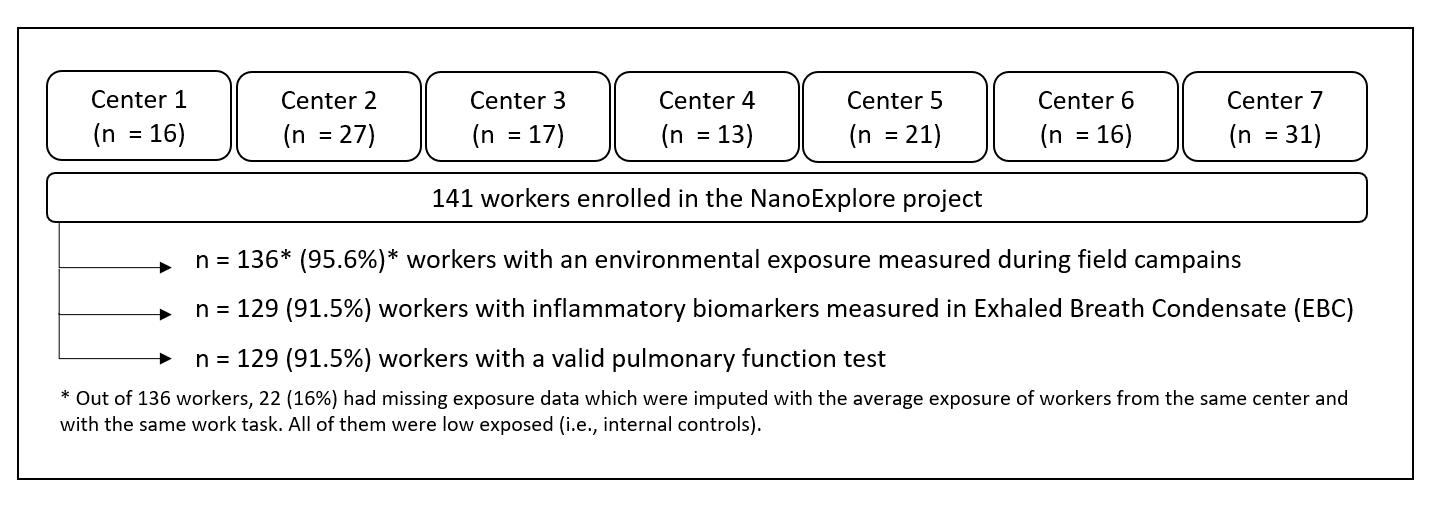
**

**Table S1.** Association between an IQR-increase of 10-year cumulative exposure to nanomaterials and lung function parameters. Estimates derived after multiple imputation.

| **Covariates** | **Exposure expressed as particle number concentration**  **(particles/cm^3^-yrs)** | | | | | | **Exposure expressed as Lung-Deposited Area Surface (****µm^2^/cm^3^-yrs)** | | | | | | | |
| --- | --- | --- | --- | --- | --- | --- | --- | --- | --- | --- | --- | --- | --- | --- |
|  | **FEV_1_** | | | **FEF_25-75%_** | | | **FEV_1_** | | | | **FEF_25-75%_** | | | |
| **GSEM including IL-10** | **OR** | **95% CI** | **p-value** | **OR** | **95% CI** | **p-value** | **OR** | **95% CI** | **p-value** | **OR** | | **95% CI** | **p-value** |  |
| 10-year cumulative exposure to nanomaterials | 2.33 | 1.30-4.19 | 0.005 | 1.99 | 1.1-3.61 | 0.023 | 3.02 | 1.52-6.00 | 0.002 | 2.55 | | 1.16-5.60 | 0.020 |  |
| Active lifestyle (inactive as reference) | 0.26 | 0.1-0.68 | 0.006 | 0.38 | 0.14-1.05 | 0.062 | 0.27 | 0.10-0.70 | 0.007 | 0.39 | | 0.14-1.07 | 0.066 |  |
| Pack-years | 1.04 | 1.01-1.09 | 0.017 | 0.99 | 0.93-1.04 | 0.561 | 1.04 | 1.00-1.07 | 0.056 | 0.99 | | 0.92-1.03 | 0.418 |  |
| **GSEM including IL-1β** | **OR** | **95% CI** | **p-value** | **OR** | **95% CI** | **p-value** | **OR** | **95% CI** | **p-value** | **OR** | | **95% CI** | **p-value** |  |
| 10-year cumulative exposure to nanomaterials | 1.91 | 1.01-3.6 | 0.048 | 1.75 | 0.92-3.35 | 0.090 | 2.51 | 1.08-5.80 | 0.033 | 2.36 | | 0.98-5.70 | 0.057 |  |
| **Active lifestyle (inactive as reference)** | 0.32 | 0.13-0.81 | 0.015 | 0.47 | 0.18-1.23 | 0.123 | 0.33 | 0.13-0.82 | 0.017 | 0.47 | | 0.18-1.25 | 0.129 |  |
| Pack-years | 1.04 | 1.003-1.08 | 0.028 | 0.98 | 0.93-1.03 | 0.444 | 1.04 | 1.001-1.008 | 0.046 | 0.98 | | 0.92-1.04 | 0.484 |  |
| **GSEM including TNF-α** | **OR** | **95% CI** | **p-value** | **OR** | **95% CI** | **p-value** | **OR** | **95% CI** | **p-value** | **OR** | | **95% CI** | **p-value** |  |
| 10-year cumulative exposure to nanomaterials | 1.81 | 0.89-3.68 | 0.100 | 1.99 | 1.04-3.79 | 0.036 | 2.19 | 1.14-4.20 | 0.020 | 2.42 | | 0.98-5.97 | 0.055 |  |
| Active lifestyle (inactive as reference) | 0.32 | 0.13-0.79 | 0.013 | 0.47 | 0.18-1.22 | 0.120 | 0.34 | 0.14-0.83 | 0.018 | 0.49 | | 0.18-1.29 | 0.126 |  |
| Pack-years | 1.04 | 1.001-1.07 | 0.044 | 0.98 | 0.93-1.03 | 0.469 | 1.03 | 1.00-1.08 | 0.062 | 0.97 | | 0.92-1.03 | 0.390 |  |

Footnote: all Odds Ratios (ORs) are derived from single-mediator Generalized Multilevel Structural Equation Models with the recruiting center and the IDs as latent variables accounting for between- and within-level variability. The ORs are adjusted by potential confounders including active/inactive lifestyle, lifetime tobacco smoking (packyears, sex, age, Body Mass Index (BMI) and ethnicity. The parameter-specific LLNs are derived from the Global Lung Function Initiative (Quanjer, 2012). Interleukins (ILs) and Tumor Necrosis Factor alpha are measured in exhaled breath condensate as pulmonary biomarker of inflammation.

**Table S2.** Association between an IQR-increase of 10-year cumulative exposure to nanomaterials (particles/cm^3^-years) and lung function parameters. Estimates derived after single imputation on the exposure variables.

| Covariates | FEV_1_ | | | FVC | | | FEV_1_/FVC | | | FEF_25-75%_ | | |
| --- | --- | --- | --- | --- | --- | --- | --- | --- | --- | --- | --- | --- |
| **GSEM including IL-10** | **OR** | **95% CI** | **p-value** | **OR** | **95% CI** | **p-value** | **OR** | **95% CI** | **p-value** | **OR** | **95% CI** | **p-value** |
| 10-year cumulative exposure to nanomaterials (particles/cm^3^-yrs) | **1.68** | **1.21-2.34** | **0.002** | 0.97 | 0.52-1.79 | 0.917 | 0.65 | 0.30-1.40 | 0.274 | **1.53** | **1.08-2.19** | **0.018** |
| Active lifestyle (inactive as reference) | **0.27** | **0.10-0.69** | **0.006** | 0.97 | 0.32-2.96 | 0.952 | **0.21** | **0.07-0.62** | **0.005** | 0.39 | 0.14-1.07 | 0.068 |
| Pack-years | **1.05** | **1.01-1.08** | **0.019** | **1.05** | **1.01-1.09** | **0.010** | 0.99 | 0.93-1.05 | 0.680 | 0.99 | 0.94-1.04 | 0.556 |
| **GSEM including IL-1β** | **OR** | **95% CI** | **p-value** | **OR** | **95% CI** | **p-value** | **OR** | **95% CI** | **p-value** | **OR** | **95% CI** | **p-value** |
| 10-year cumulative exposure to nanomaterials (particles/cm^3^-yrs) | **1.52** | **1.08-2.14** | **0.018** | 0.79 | 0.39-1.60 | 0.510 | 0.73 | 0.37-1.41 | 0.343 | 1.43 | 0.98-2.07 | 0.063 |
| Active lifestyle (inactive as reference) | **0.32** | **0.13-0.79** | **0.014** | 1.08 | 0.35-3.29 | 0.894 | **0.29** | **0.11-0.81** | **0.017** | 0.47 | 0.18-1.23 | 0.124 |
| Pack-years | **1.040** | **1.004-1.070** | **0.028** | **1.05** | **1.01-1.10** | **0.013** | 1.00 | 0.95-1.05 | 0.890 | 0.98 | 0.93-1.03 | 0.444 |
| **GSEM including TNF-α** | **OR** | **95% CI** | **p-value** | **OR** | **95% CI** | **p-value** | **OR** | **95% CI** | **p-value** | **OR** | **95% CI** | **p-value** |
| 10-year cumulative exposure to nanomaterials (particles/cm^3^-yrs) | **1.470** | **1.002-2.150** | **0.049** | 0.68 | 0.28-1.64 | 0.387 | 0.67 | 0.35-1.28 | 0.227 | **1.52** | **1.04-2.20** | **0.029** |
| Active lifestyle (inactive as reference) | **0.31** | **0.13-0.77** | **0.012** | 1.03 | 0.34-3.14 | 0.953 | **0.29** | **0.11-0.82** | **0.019** | 0.47 | 0.18-1.22 | 0.122 |
| Pack-years | **1.040** | **1.001-1.070** | **0.046** | **1.05** | **1.01-1.11** | **0.020** | 1.00 | 0.95-1.05 | 0.951 | 0.98 | 0.93-1.03 | 0.471 |

Footnote: all Odds Ratios (ORs) are derived from single-mediator Generalized Multilevel Structural Equation Models with the recruiting center and the IDs as latent variables accounting for between- and within-level variability. The ORs are adjusted by potential confounders including active/inactive lifestyle, lifetime tobacco smoking (packyears, sex, age, Body Mass Index (BMI) and ethnicity. The parameter-specific LLNs are derived from the Global Lung Function Initiative (Quanjer, 2012). Interleukins (ILs) and Tumor Necrosis Factor alpha are measured in exhaled breath condensate as pulmonary biomarker of inflammation.

**Table S3.** Association between an IQR-increase of 10-year cumulative exposure to nanomaterials (LDSA, i.e., µm^2^/cm^3^-years) and lung function parameters. Estimates derived after single imputation on the exposure variables.

| Covariates | FEV_1_ | | | FVC | | | FEV_1_/FVC | | | FEF_25-75%_ | | |
| --- | --- | --- | --- | --- | --- | --- | --- | --- | --- | --- | --- | --- |
| **GSEM including IL-10** | **OR** | **95% CI** | **p-value** | **OR** | **95% CI** | **p-value** | **OR** | **95% CI** | **p-value** | **OR** | **95% CI** | **p-value** |
| 10-year cumulative exposure to nanomaterials (µm^2^/cm^3^-years) | **2.11** | **1.36-3.27** | **0.001** | 1.13 | 0.59-2.19 | 0.708 | 0.65 | 0.30-1.41 | 0.274 | **1.86** | **1.15-3.03** | **0.012** |
| Active lifestyle (inactive as reference) | **0.27** | **0.11-0.70** | **0.007** | 0.97 | 0.32-2.98 | 0.708 | **0.23** | **0.08-0.69** | **0.008** | 0.40 | 0.15-1.09 | 0.073 |
| Pack-years | 1.03 | 1.00-1.07 | 0.062 | **1.05** | **1.01-1.09** | **0.018** | 1.00 | 0.94-1.05 | 0.779 | 0.98 | 0.92-1.03 | 0.400 |
| **GSEM including IL-1β** | **OR** | **95% CI** | **p-value** | **OR** | **95% CI** | **p-value** | **OR** | **95% CI** | **p-value** | **OR** | **95% CI** | **p-value** |
| 10-year cumulative exposure to nanomaterials (µm^2^/cm^3^-years) | **1.95** | **1.15-3.32** | **0.014** | 0.90 | 0.46-1.75 | 0.750 | 0.59 | 0.27-1.28 | 0.178 | **1.82** | **1.03-3.23** | **0.041** |
| Active lifestyle (inactive as reference) | **0.32** | **0.13-0.80** | **0.014** | 1.09 | 0.36-3.31 | 0.886 | **0.30** | **0.11-0.82** | **0.018** | 0.47 | 0.18-1.23 | 0.125 |
| Pack-years | 1.04 | 1.00-1.07 | 0.051 | **1.05** | **1.01-1.09** | **0.018** | 1.00 | 0.94-1.05 | 0.905 | 0.98 | 0.92-1.03 | 0.494 |
| **GSEM including TNF-α** | **OR** | **95% CI** | **p-value** | **OR** | **95% CI** | **p-value** | **OR** | **95% CI** | **p-value** | **OR** | **95% CI** | **p-value** |
| 10-year cumulative exposure to nanomaterials (µm^2^/cm^3^-years) | **1.75** | **1.16-2.65** | **0.008** | 0.78 | 0.33-1.83 | 0.569 | 0.69 | 0.35-1.28 | 0.222 | **1.81** | **1.01-3.26** | **0.048** |
| Active lifestyle (inactive as reference) | **0.32** | **0.13-0.80** | **0.014** | 1.1 | 0.36-3.36 | 0.87 | **0.29** | **0.11-0.82** | **0.019** | 0.48 | 0.18-1.28 | 0.142 |
| Pack-years | 1.03 | 0.99-1.07 | 0.065 | **1.05** | **1.01-1.09** | **0.027** | 1.00 | 0.94-1.05 | 0.996 | 0.98 | 0.92-1.03 | 0.406 |

Footnote: all Odds Ratios (ORs) are derived from single-mediator Generalized Multilevel Structural Equation Models with the recruiting center and the IDs as latent variables accounting for between- and within-level variability. The ORs are adjusted by potential confounders including active/inactive lifestyle, lifetime tobacco smoking (packyears, sex, age, Body Mass Index (BMI) and ethnicity. The parameter-specific LLNs are derived from the Global Lung Function Initiative (Quanjer, 2012). Interleukins (ILs) and Tumor Necrosis Factor alpha are measured in exhaled breath condensate as pulmonary biomarker of inflammation.

**Table S4.** Association between an IQR-increase of 10-year cumulative exposures to nanomaterials and lung function parameters, accounting for the anti-pro inflammatory ratio (IL-10/TNF-α). Estimates derived after single imputation on the exposure variables.

| **Covariates** | **FEV_1_** | | **FVC** | | **FEV_1_/FVC** | | | **FEF_25-75%_** | | | |
| --- | --- | --- | --- | --- | --- | --- | --- | --- | --- | --- | --- |
| **GSEM including anti-pro ratio** | **OR (95% CI)** | **p** | **OR (95% CI)** | **p** | **OR (95% CI)** | **p** | | **OR (95% CI)** | | **p** |  |
| 10-yrs cumulative exposure to **particle number concentration** | **1.46 (1.02-2.15)** | **0.039** | 0.74 (0.33-1.71) | 0.491 | 0.76 (0.42-1.38) | 0.365 | | 1.43 (1.00-2.05) | | 0.052 |  |
| Active lifestyle (inactive as reference) | **0.29 (0.12-0.73)** | **0.009** | 1.03 (0.34-3.10) | 0.969 | **0.25 (0.09-0.71)** | **0.009** | | 0.47 /0.18-1.25) | | 0.131 |  |
| Pack-years | **1.04 (1.00-1.07)** | **0.041** | **1.05 (1.01-1.10)** | **0.016** | 1.00 (0.94-1.05) | 0.776 | | 0.98 (0.93-1.03) | | 0.440 |  |
| **Biomarker change in response cumulative exposure:** | **β (95% CI)** | **P** | **β (95% CI)** | **P** | **β (95% CI)** | **P** | | **β (95% CI)** | | **p** |  |
| IL-10/TNF-α | **-1.23 (-2.14;-0.32)** | **0.008** | **-1.23 (-2.14;-0.32)** | **0.008** | **-1.23 (-2.14;-0.32)** | **0.008** | **-1.23 (-2.14;-0.32)** | | **0.008** | |  |
| **GSEM including anti-pro ratio** | **OR (95% CI)** | **P** | **OR (95% CI)** | **P** | **OR (95% CI)** | **P** | | **OR (95% CI)** | | **p** |  |
| 10-yrs cumulative exposure to LDSA | **1.72 (1.17-2.53)** | **0.006** | 0.84 (0.37-1.91) | 0.670 | 0.76 (0.42-1.41) | 0.391 | | **1.81 (1.02-3.2)** | | **0.043** |  |
| Active lifestyle (inactive as reference) | **0.30 (0.12-0.75)** | **0.010** | 1.06 (0.34-3.25) | 0.925 | **0.25 (0.09-0.70)** | **0.008** | | 0.46 (0.17-1.23) | | 0.120 |  |
| Pack-years | 1.03 (1.00-1.07) | 0.075 | **1.05 (1.01-1.09)** | **0.022** | 1.00 (0.94-1.05) | 0.807 | | 0.98 (0.93-1.04) | | 0.459 |  |
| **Biomarker change in response cumulative exposure:** | **β (95% CI)** | **p** | **β (95% CI)** | **p** | **β (95% CI)** | **p** | | **β (95% CI)** | | **p** |  |
| IL-10/TNF-α | **-1.28 (-2.35;-0.20)** | **0.020** | **-1.28 (-2.35;-0.20)** | **0.020** | **-1.28 (-2.35;-0.20)** | **0.020** | | **-1.28 (-2.35;-0.20)** | | **0.020** |  |

Footnote: all Odds Ratios (ORs) are derived from single-mediator Generalized Multilevel Structural Equation Models with the recruiting center and the IDs as latent variables accounting for between- and within-level variability. The ORs are calculated for an IQR-increase of cumulative exposure to nanomaterials (10 years) and are adjusted by potential confounders including active/inactive lifestyle, lifetime tobacco smoking (packyears), sex, age, Body Mass Index (BMI) and ethnicity. The parameter-specific Lower Limit of Normal (LLN) are derived from the Global Lung Function Initiative (Quanjer, 2012) to express each respiratory parameter as below or above the fifth percentile (or a z-score < − 1.64) of the GLI SRE distribution. Interleukins (ILs) and Tumor Necrosis Factor alpha are measured in exhaled breath condensate as pulmonary biomarker of inflammation.

**Table S5**. Description of nanomaterial exposure in exposed workers

| Type of nanomaterial declared | Company-declared exposure | Participant self-declared exposure |
| --- | --- | --- |
| Ag nanoparticles | 4 (4.1) | -- |
| Au nanoparticles | 4 (4.1) | -- |
| Biodegradable polymers | 6 (6.2) | -- |
| Black carbon | -- | 14 (14.4) |
| CaCO3 | 17 (17.5) | -- |
| Carbon nanotubes | 8 (8.2) | 4 (4.1) |
| Cellulose | 9 (9.3) | -- |
| FeO | 11 (11.3) | -- |
| Methacrylate / acrylate | 6 (6.2) | -- |
| SiO2 | 16 (16.5) | 29 (29.9) |
| TiO2 | 15 (15.5) | 23 (23.7) |
| Other | -- | 17 (17.5) |
| No exposure | -- | 24 (24.7) |
| Not known | -- | 29 (29.9) |

**References**

1. Quanjer PH, Stanojevic S, Cole TJ, Baur X, Hall GL, Culver BH, et al. Multi-ethnic reference values for spirometry for the 3–95-yr age range: the global lung function 2012 equations. Eur Respir J [Internet]. 2012 Dec 1;40(6):1324–43. Available from: https://erj.ersjournals.com/content/40/6/1324
